# Supplementary material for: Incidence of critical events in the post-anesthesia care unit at a resource-limited setting in Debre Markos, Northwest Ethiopia
Source: PLoS One. 2026 Mar 24;21(3):e0345873. doi: 10.1371/journal.pone.0345873 (PMC13012457; doi:10.1371/journal.pone.0345873)
Supplement: Attachment — Submitted filename: Response to Reviewers.docx [file pone.0345873.s003.docx]

**Responses to editors' and reviewers' comment**

First of all, we would like to acknowledge the reviewers and editorial team for giving your time to review this manuscript. Then we have tried to reflect our responses as follow for reviewers and editors according to your concerns.

I. **Editors**

1. Please ensure that your manuscript meets PLOS ONE's style requirements, including those for file naming. The PLOS ONE style templates can be found at. **Response**: we have tried to be in line with PLOS ONE style.

https://journals.plos.org/plosone/s/file?id=wjVg/PLOSOne_formatting_sample_main_body.pdf and https://journals.plos.org/plosone/s/file?id=ba62/PLOSOne_formatting_sample_title_authors_affiliations.pdf

2. Please amend the manuscript submission data (via Edit Submission) to include author Eniyew Fenta.

3. Please amend your authorship list in your manuscript file to include author Enyew Fenta Mengistu. **Response**: It’s been corrected.

4. Please remove your figures from within your manuscript file, leaving only the individual TIFF/EPS image files, uploaded separately. These will be automatically included in the reviewers’ PDF. **Response**: corrected.

5. We are unable to open your Supporting Information file [PACU complication.sav final.sav]. Please kindly revise as necessary and re-upload. **Response**: we re-uploaded the final version we used for analysis.

6. Please include captions for your Supporting Information files at the end of your manuscript, and update any in-text citations to match accordingly. Please see our Supporting Information guidelines for more information: http://journals.plos.org/plosone/s/supporting-information.

If the reviewer comments include a recommendation to cite specific previously published works, please review and evaluate these publications to determine whether they are relevant and should be cited. There is no requirement to cite these works unless the editor has indicated otherwise. **Response**: appropriate measurements were taken based on reviewers concern.

Additional Editor Comments:

The abstract section does not need to include a separate "Objective" heading, as the objective is already part of the background. Please revise and proofread the entire manuscript carefully, as there are numerous grammatical errors and typographical issues. Ensure consistency in terminology, especially regarding the use of "overweight" and "obesity," and avoid casual or interchangeable language. **Response**: as much as possible we tried to correct the issues you mentioned above.

Instead of stating, “Today, surgical procedures are accepted as one of the most important therapeutic methods in all societies worldwide,” consider rephrasing to: “Nowadays, surgical procedures are widely recognized as essential therapeutic interventions across all societies.” **Response**: it’s corrected.

Avoid redundancy and unnecessary detail. For example, the following sentence should be revised for clarity and brevity:

“A post-anesthesia care unit, often abbreviated as PACU and sometimes referred to as post-anesthesia recovery (PAR), is a vital part of hospitals. It is an area, normally attached to operating room suites, designed to provide care for patients recovering from general anesthesia, regional anesthesia, or local anesthesia.” **Response**: it’s corrected

This should be simplified and written more precisely.

Remove the separate headings for “General” and “Specific Objectives” and instead integrate the study aim into the final paragraph of the introduction. Please refer to the PLOS ONE journal format and review previously published articles to guide your revisions.

The “Methods and Materials” section requires substantial language editing. For example, the sentence:

“Patients who were transferred directly from the operation room to an intensive care unit or ward excluded in the study,” should be rephrased and justified, as major surgical procedures are often followed by direct transfer to ICU or ward, which may influence the incidence of critical events. **Response**: our focus is just to investigate the immediate post-operative critical events which means in the PACU. Otherwise if we included those patients transferred to ICU and ward, we belied that the incidence and associated factors could be changed from the PACU. Because the care given as well as the ratio of health care provider to patients in the ICU and ward if different from PACU, this affects our study. That is why we left ICU and ward patients.

Although the authors state that a 50% prevalence was used due to the lack of similar studies in Ethiopia, there is in fact a related study with comparable findings. Please revise the justification accordingly. **Response**: yes there are some studies in Ethiopia but the setup of these studies much better that our study area. Based on our previous observations, we perceived the PACU standard of our study hospital was poor. We discussed that to use 50% prevalence to investigate the situation with better sample size.

Explain why seven data collectors and six supervisors were involved, as this may introduce variability and bias. Revise the data collection section to improve clarity and eliminate ambiguity. **Response**: why we increased number of data collectors and supervisors, it is due to the nature of the study since we included all cases means emergencies and electives we have to collect all cases at nights, working days, and holidays. To do this we need relatively large number of data collectors and supervisors. It was difficult to small numbers of data collectors and supervisors. That is why we include relatively large numbers.

Clarify why the pre-test was conducted at the same institution where the study took place. **Response**: we consult experts and what they told us, you can do pretest in the same area but don’t included in the analysis. During our study period, there was active war thus both the internet and roads were locked. Therefore, we couldn’t do pretest in other institutions.

The terminology regarding outcomes is inconsistent. Terms such as “PACU complications,” “critical events,” and “critical incidents” are used interchangeably. Please choose a single term and use it consistently throughout the manuscript. **Response**: corrected

In the study population, clarify the age range included. If the study spans a wide range (e.g., 3 to 86 years), consider whether this is clinically appropriate, given that complication rates and types likely differ across age groups. **Response**: what we focused is just what the status of critical events in the PACU is. We want to investigate any types of critical events irrespective of age, sex, types of surgeries and the like. But we analyzed whether these factors have association or not with critical events. We considered age as independent factor and found that age was not associated with critical events.

Clarify whether your source population includes all surgical patients or only those undergoing major surgery. Also, assess whether including procedures performed under sedation in both major and minor ORs introduces clinical heterogeneity. **Response**: rephrased; the source population is all surgical patients who revived any types of anesthesia in the Major operation room.

Table 2 needs revision, as some values (e.g., total sum of critical events by ASA class) exceed 100%. Verify and correct the data presentation. **Response**: we have checked and may be the table is ambiguous but the proportion of critical events in subgroups (%) is the ratio of patients with critical events to the total number of patients in specific groups (eg in age below 15 years 18/53*100 =34%). This values can’t give 100%. These are just ratios in specific groups.

Patients undergoing general, regional, or sedative anesthesia represent clinically distinct groups. Consider whether it is appropriate to analyze them together when measuring the incidence of postoperative critical events. **Response**: we don’t believe that it is feasible to study the incidence and types of critical events by dividing by different types of anesthesia. It is better to check whether these types of anesthesia have association or not. What we do is just analyzed whether these types of anesthesia have association with critical events or not.

Further comments on the results and discussion sections will follow once the above major issues have been addressed.

**II: Reviewers**

Reviewer #1: Abstract; methodology (rephrase); **Response**: The whole paper including abstract has been re-written

Background; paragraph 2: On the other hand, delayed effects of different anesthetic agents and muscle relaxants affects negatively the natural ability of the body to maintain physiological balance, which finally causes post-anesthetic critical incidents (critical events). (cite/ find reference): **Response;** we have cited

last paragraph: Therefore the finding of this study will contribute in minimizing the doubt regarding the type of factors and common post-operative critical incidents. (rephrase); **Response:** Based on your suggestions we review grammatical and clarity issues carefully and it is rephrased

Methodology; data collection; (rephrase)**; Response:** it is rephrased

Results: repeated several times in the text; **Response**: we tried to avoid unnecessary redundant words in the whole text.

Discussion: More of literature review. Discuss why you got the results. Highlight the limitations and explain why eg: 2 hour delay, Improper passing over etc. **Response**: we tried to address all issues.

Reviewer #2: Well done. I love your research & how it was done. However, there are a couple issues I have with your work: **Response**: Thank you very much dear reviewer for your appreciation.

There are a lot of grammatical errors and poorly constructed sentences, especially in the abstract and background sections. Also, your work did not include If day cases or people already on admission had an effect on critical events in PACU. **Response**: based on your suggestion we have reviewed carefully the issues of grammar and bad sentence constructions and we have taken measurements to make the study readable for scholars. Based on the protocol of the study hospital all patients who received anesthesia should be admitted in the recovery (PACU) except those patients directly sent to ICU. Although we have written ‘those patients directly transferred from operation room to ward and ICU as exclusion, but in practice there was no patient directly transferred to ward rather there were to ICU. Therefore, based on the study institution protocol all cases should be admitted to PACU before they discharged to ward or home. So, no cases missed once they exposed for anesthesia. We removed the word ‘ward’ in the manuscript since there were no patient transferred in this sentence ‘Patients who were transferred directly from the operation room to **ward** excluded in the study’.

Besides, I'd have loved it personally if the chart at the end of the work is brought into the result section. I'll have also loved if discharge scoring systems in PACU were examined in relation to critical incidence. **Response**: Thank you dear reviewer for your suggestions. Although we didn’t assess the scoring system of discharge but we haves assessed whether critical incidents prolong the timing of discharge and the result indicated that it significantly prolongs the PACU stay time. Scholars reported that prolonged stay in the PACU has economic burdens both for patents and hospitals.

Reviewer #3: Reviewer Report for Manuscript PONE-D-25-27675

Summary :

This prospective study on PACU complications in a resource-limited Ethiopian hospital addresses a significant knowledge gap. While the topic is important and the design has strengths, major methodological, statistical, and reporting flaws preclude acceptance. Extensive revisions and re-analysis are essential.

Major Concerns :

1. Methodological Rigor :

- Sample size calculation using 50% prevalence lacks justification. Recalculate using literature-based estimates (e.g., 20-35%) and perform post-hoc power analysis. **Response**: why we didn’t use previous studies is, we believed the setup of our study area far different than the others. Based on our previous observations, the study area lacks ether local or international guidelines like WHO, ASA… etc. therefore, our expectation was the incidence rate greater than we found in this study. So, why we used 50% prevalence is just to investigate critical events with larger sample size.

- Critical event definitions are inconsistent and unvalidated (e.g., SpO₂<90% for >3 minutes; Clavien-Dindo applied to PACU without validation). Standardize terminology ("critical events" vs. "complications") and align with WHO/ANZCA guidelines. Report inter-rater reliability (κ-statistic). **Response**: we used critical events from different studies as we cited and we didn’t used Clavien-Dindo’s report for data collection rather we just used for comparison once we analyzed. It’s to know in which grade of Clavien-Dindo grading our finding was lied. Regarding terminology, ANZCA and many literatures used both terms but for preventable serious sudden complications used either critical incidents or events. Therefore, in the PACU the types of complications happened are most of the time are serious unless managed early. We made consistent the terms and used the term critical events.

- Data collection gaps : No standardized discharge criteria (e.g., Aldrete score), and missing data strategy unreported. Address "na" entries in complication timing/type (13% of events). **Response**: discharge criteria was not in our study objectives. Our objective was to study critical events. But as a secondary objective we assessed whether there was association between critical events and fulfillment of patients to discharge criteria at 2 hours using Aldrete score. As a study we used Aldrete score but the study institution hadn’t it. Regarding the missing data management, although it is possible to use different strategy of missing data management like using mean, median, or mode but we authors agreed to reject the data which contains missing content and replace with other data until the calculated data fulfilled to improve the accuracy of data. Luckily we had no any missing content in our data.

2. Statistical Validity :

- Regression models are inadequately developed: **Response**: we have discussed with biostatisticians after your suggestions and they gave us binary logistic regression is appropriate model to analyze our data. We checked all the assumptions of logistic regression and it fitted.

- Age retained despite non-significance (p=0.31); no justification for variable inclusion. : **Response**: sorry for making the table ambiguous. But the COR p-value (bivariable analysis) of age was below 0.2 and the p-value set in the table 5 is only for AOR

- Multicollinearity unchecked (e.g., ASA-BMI-duration correlation likely). **Response**: we have checked the assumption of logistic regression and the data fulfils the assumption with VIF value of below 2.3, tolerance value greater than 0.4, and inter-correlation of spearman correlation value below 0.7. Although logically BMI affects ASA status in our study the statistics indicate that there is no correlation between variables.

- Full bivariable results omitted; sensitivity analyses missing. **Response**: we left bivarible result from the final table to avoid the condensed table. But after your suggestion we observed as it is ambiguous and we incorporated the Crude odd ratio in table 5. Table 5 contains those variables that fulfill COR p-value of < 0.2 and then run for multivariable logistic regression.

- Incorrect incidence reporting :

- Cardiovascular events: 42% of *events* reported without *patient-level* incidence (21.3% of patients). **Response**: corrected

- Table 2 mislabels subgroup *incidence* (e.g., 69.5% in ≥65yrs) as "proportions," lacking 95% CIs. **Response**: table 2 to table 4 are frequency tables and the labeling is corrected and the frequency of critical events in each sub groups were inserted.

- Data quality issues :

- Pediatric patients categorized with adult BMI. **Response**: we used the BMI on Age- and Sex-Specific Charts of CDC for children then we included the underweight in <18.5 group, the healthy weight in 18.5-24.9, and overweight & obese in >24.9 groups. But as you said it is not clear for readers and we have changed the way of reporting. We operationalize underweight, healthy weight and overweight then all patients categorized in either of the three groups whether pediatrics or Adults. **Underweight: the BMI below 18.5 kg/m2 for adults and BMI < 5th percentile for children. Healthy weight: BMI from 18.5 to 24.9 kg/m2 for adults and BMI ≥ 5th and < 85th percentile for children. Overweight: BMI ≥ 25 kg/m2 for adults and BMI ≥ 85th percentiles for children. Obese and sever obese are included under overweight for the study purpose.**

- Inconsistent coding ("femal," "secere," variable "na" rates). **Response**: we tried to make it consistent throughout the paper

3. Reporting & Interpretation :

- Results :

- No CONSORT diagram for patient inclusion. **Response**: we have inserted the flow chart

- Table 5: Wide CI for ASA4 (AOR=11.75, 95% CI: 2.76-50.03) suggests instability; unaddressed. **Response**: why we found the wide CI might be due to low number of ASA4 patents compared to the other and it mightn’t happened if the sample size was larger.

- Discussion :

- Poor contextualization of high complication rates (37.9%) within resource limitations (e.g., 54.4% complications without nurse training). **Response**: 54.4% in table3 is not the complication rate rather it is the proportion of patients with complication within the sub group in which patients who were attended by nurses who had no PACU training (118*100/217).

- War-related caseload reduction ignored in generalizability. **Response**: as we mentioned in the paper, during the study period there was active war and mostly the access of transportation to health institutions was limited due to main roads were closed. Due to this reason the case flow per day roughly reduced from 15 to 6. This extended our data collection period. Otherwise we didn’t believe that it affect the incidence of critical events.

- Limitations : Single-center design, observer bias, and selection bias (ICU-excluded patients) inadequately discussed. **Response**: we agreed with your idea regarding limitations and we will mention in our paper to be improved in the future. But regarding ICU patients, why we excluded ICU patients, they were sent directly to the ICU from the operation room. Therefore, once patients sent to ICU we believe that the incidence & risk factors should be studied independently. The care given to patients, professionals to patient ratio, and other factors make different the incidence and associated factors in ICU compared to PACU.

Essential Revisions :

1. Statistical Re-analysis:

- Report patient-level incidence with 95% CIs for *all* complications (e.g., "21.3% [17.5–25.7%] of patients had cardiovascular events"). **Responded above**

- Re-run regressions: Provide full bivariable results, check multicollinearity (VIF), validate models (ROC-AUC), and conduct sensitivity analyses. **Response**: biostatisticians suggested us validate models (ROC-AUC) and sensitivity analyses are not mandatory for this study since the study is association-focused.

- Post-hoc power analysis for key outcomes. **Response**: We have consulted biostatisticians and they suggested us as it does not add real information beyond the confidence interval and p-value. They told us ‘if your test was significant, post-hoc power will almost always look “high”; if not significant, it will look “low.” Therefore, we omitted the analysis.

2. Methodological Corrections :

- Correct pediatric BMI categorization. **Responded above**

- Define missing data handling (e.g., "Multiple imputation used for 13.1% missing complication timings"). **Response**: after your suggestion we have rechecked the hard data collection paper and the SPSS data we used for final analysis. There was not missed data of complication timing. May be we submitted the draft copy of the data. We will submit the final correct data to PLOS ONE

- Justify event definitions and validate Clavien-Dindo for PACU. **Response**: the word event in the paper used interchangeably with critical events which is mentioned in the operational definitions. Since the Clavien-Dindo grading system isn’t used as data collection tool, we didn’t believe to validate it. Just we used as a comparator of our findings. But due to the journal recommendation we used the term ‘critical event’ throughout the manuscript.

3. Reporting Compliance :

- Add 95% CIs to *every* incidence rate (Abstract, Results, Tables 2–5). **Response**: we amended the tables labeling and we added the frequency of critical events in each subgroup.

- Relabel Table 2: " Incidence of critical events, % (95% CI) " with footnote: "*% of subgroup patients with ≥1 event*." **Response**: almost all subgroups have critical events more than 1 except orthopedic surgery group. Therefore, mentioning with footnote seems not that much important for readers. If some of subgroups developed, it would indicate readers which subgroup is with different types of complication.

- Ethical committee approval number and data repository/access details (PLOS ONE requirement). **Response**: we obtained ethical approval from the college ethical review committee and we submitted the data to PLOS ONE.

4. Contextual Enhancement :

- Discuss war impact on caseload/complications. **Response**: we mentioned the impact of war on case load as we mentioned above. Main roads were locked for nearly a year and it reduced the patient flow to the study hospital.

- Contrast findings with LMIC studies. **Response**: we tried to compared

- Propose actionable interventions (e.g., nurse training program budget impact). **Response**: tried to propose based on our findings.

Long-term Recommendations: **Response**: we provided our recommendations by incorporating your suggestion
