# Supplementary material for: Incidence of critical events in the post-anesthesia care unit at a resource-limited setting in Debre Markos, Northwest Ethiopia
Source: PLoS One. 2026 Mar 24;21(3):e0345873. doi: 10.1371/journal.pone.0345873 (PMC13012457; doi:10.1371/journal.pone.0345873)
Supplement: Attachment — Submitted filename: Response_to_Reviewers_auresp_2.docx [file pone.0345873.s004.docx]

First, we would like to thank the reviewers and the editorial team for taking the time to review our manuscript. We have addressed your concerns as follows in our responses to the reviewers and editors.

**Additional Editor Comments:**
In the abstract section
In methods of abstract section please revise it in this way “An institution-based prospective cross-sectional study was conducted from June 1, 2024, to September 30, 2024.” Revise as follows “Analysis was conducted using bivariable and multivariable logistic regression as needed.” **Response**: corrected
In the results section, you stated that "Out of 422 operations, 37.9% of patients developed about 214 complications." Please consider rephrasing this statement for clarity.
In the conclusion, revise as follows: “Patients who had intraoperative complications, higher ASA status, unhealthy body weight, and those who received anesthesia for a prolonged duration were relatively at higher risk of developing critical events.” Please replace the term "unhealthy body weight" with the appropriate term using BMI. **Response**: corrected

Change the heading title from "Background" to "Introduction" at the end of the abstract; it should begin as "Introduction," not "Background." **Response**: corrected

In the introduction section, please include references that support the evidence you are using as much as possible. I've noticed that some statements have only one or two citations, despite being quite broad. **Response**: we considered your suggestions

The language and grammar have been significantly improved; however, it still needs revision. Please read it carefully line by line and use appropriate transition terms to connect each statement throughout the entire document. **Response**: we considered your suggestions

Revise as stated “In developing countries, including Ethiopia, most PACU setups do not meet WHO standards, and some hospitals lack a PACU altogether, sending patients directly from the operating room to the ward.” **Response**: corrected

You mentioned that "Although several studies have been published on complications in the PACU, there is limited information in Africa." However, you need to provide a reference for this statement. **Response**: we have cited based on your recommendation

Please refrain from providing detailed lists of independent and dependent variables. Instead, use statements to describe them. **Response**: corrected

Please provide details on how the data were transitioned from bivariate to multivariate analysis, as well as how multicollinearity was assessed in the data analysis section. **Response**: corrected

Please provide details in the limitations section. For example, various types of surgical procedures and significant critical event types were assessed, and a wide range of age groups was included. It is recommended that specific research focusing on particular types of surgery be conducted. **Response**: corrected

**Reviewer #2**: This research topic is very interesting and important also. I'm also happy with the improvement in the review of the article as compared to the initial draft, especially as regards grammar and construct. I appreciate the correlations between critical incidents and ASA classification, trained personnel, BMI and other criteria. I also appreciate the "Recommendation" section because the article did outline the need for a proper PACU handover criteria and training of PACU personnel to help with prevention and management of complications.

However, I'll like to query the choice of words and terminologies such as Tachyarrythmia and Bradyarrythmia over tachycardia and bradycardia. Hypertension with Systolic Blood Pressure >180mmHg is according to what criteria? **Response**: we used the definition set in the anesthesia book of Morgan & Mikhail’s Clinical Anesthesiology, 7th ed. McGraw-Hill, 2022. And also we used previous studies cited in our paper. And we corrected the terminologies as your suggestions to tachycardia and bradycardia.
When patients were finally discharged from PACU, what discharge criteria was used? **Response**: we assessed whether there was association between critical events and fulfillment of patients to discharge criteria at 2 hours using Aldrete score. As a study we used Aldrete score but the study institution hadn’t it. Whether professionals planned to discharge patients from PACU or not we assessed all patients after 2 hours of waiting in PACU. But in practical most of patients discharged from PACU after more than 5 hours waiting due to the absence of discharge protocol in the institution.

Besides, your study did not account for any difference in critical incidence between Day cases and in patients, if there are any. **Response**: although our inclusion criteria allows to include day cases in our study, but there were no day-case procedures in our study since the hospital had no day-case schedule till end of our study period.

Lastly, a few grammar and construct adjustments could be made to perfect the article. Nonetheless, it's a work well done! **Response**: thank you for your appreciation dear reviewer for your constructive comments to make this article an interesting and we tried to correct the grammar and other concerns.

**Reviewer #3**: General Comments
This is an important prospective study addressing perioperative safety in a resource-limited setting. The topic is highly relevant, particularly given the paucity of data on PACU outcomes in sub-Saharan Africa. The revised version has improved substantially; however, there remain issues in clarity, structure, reporting, and methodological detail that should be addressed before acceptance. Below, I provide detailed comments for the authors and editorial team.

Major Concerns
Abstract

The sentence “A P-value <0.05 was considered statistically significant” should be removed from the abstract; such technical details belong in the Methods. **Response**: corrected

The conclusion is vague: “A significant number of patients suffered…”. Please quantify (“Approximately 38% of patients experienced ≥1 critical event, mainly cardiovascular and respiratory complications.”). **Response**: we have corrected based on your suggestions

Background

The abbreviation ASA is used without definition. Please spell out in full at first mention (American Society of Anesthesiologists physical status classification), then abbreviate. **Response**: we have corrected.

Methods

Independent/Dependent Variables: These are listed as bullet points (p. 5). For PLOS ONE style, rephrase into sentences/paragraphs (e.g., “Independent variables included age, sex, ASA status, BMI, type of anesthesia…”). **Response**: it’s corrected based on your suggestions.

Statistical Model Fit: The manuscript mentions Omnibus and Hosmer–Lemeshow tests but does not explain them. Please describe their purpose and null hypotheses (Omnibus: H₀ = no predictors improve the model; Hosmer–Lemeshow: H₀ = model fits observed data). Interpret the reported p-values (e.g., p=0.085 indicates no evidence of poor fit). **Response**: we have corrected based on your suggestions.

Adjustment Strategy: Clarify that only variables with p<0.20 in bivariable analysis were retained for multivariable regression, and state explicitly that other covariates were excluded. **Response**: we clarified in this way ‘Only variables with a p-value < 0.20 in the bivariable analysis were included in the multivariable regression model, while covariates with p ≥ 0.20 were excluded’.

Ethics: The ethics section mentions approval but no code/number. Please provide the approval code, or explicitly state that the committee did not issue one. **Response**: we have ethical approval letter with the code of R/C/S/D/357/01/18. And, if we are requested we can attach the letter. We corrected and mentioned in ethics part.

Clavien–Dindo Grades: Consider a separate regression analysis restricted to Grade III–V complications to identify predictors of severe, life-threatening events. **Response**: we have faced difficulties to analyses the association between dependent variables and covariates in the samples who have Clavien–Dindo class III and IV. It is not fulfilling the assumption (large covariates with small sample).

Results – Tables

Table 2–4: The column header “Proportion of critical events in subgroups” should be replaced with “Incidence of critical events (% with ≥1 event, 95% CI)”. Confidence intervals should be added. **Response**: corrected

Narrative Clarity: Avoid vague terms like “nearly equal” (p. 11); report actual numbers/percentages. **Response**: corrected.

Event Reporting: For each complication (hypotension, shock, arrhythmia, etc.), present both counts and percentages (e.g., “41 cases of hypotension, 19.2%”). **Response:** corrected

Nurse Training Comparison: Where differences are noted (trained vs. untrained), present percentages and statistical results (p-values/AOR) in the text, not only in tables. **Response**: we have mentioned it in text addition to the table in page 13.

Table 5:

Add a column for p-values corresponding to CORs. **Response**: corrected

Clarify which covariates were included in AOR adjustment. **Response**: we have mentioned variables which included in AOR adjustment in text part.

Figures – Flow Diagram

The boxes require alignment correction for readability.

Excluded patients are not quantified. Please add the total n of excluded patients with reasons.

The n = 422 included label should be placed adjacent to “Patients included in the study,” not before.

The final node should branch into two groups: “Patients with ≥1 critical event (n = …)” and “Patients without critical events (n = …)”. This would better communicate study outcomes.

**Response**: re-adjusted

Limitations

The limitations section should note that the study only assessed immediate PACU complications and could not capture delayed but clinically important events such as pulmonary thromboembolism (PTE). Acknowledge that this is a serious limitation of the PACU-only timeframe. **Response**: we accept all of your comments as it is. And we accept this very important comment also and we incorporated in the limitation section.

Minor Concerns
Replace the phrase “post-anesthesia complications also known as critical incidents” with more consistent terminology (“critical events”) throughout. **Response**: rephrased

Ensure that percentages are consistently reported alongside raw numbers across Results and Discussion. **Response**: we have checked it

Revise wording such as “nearly equal” or “not surprising” with precise, scientific expressions. **Response**: we have corrected.

Flow and grammar should be further polished (e.g., “a P-value <0.05 was consider” → “considered”). **Response**: corrected
