# Supplementary material for: Incidence of critical events in the post-anesthesia care unit at a resource-limited setting in Debre Markos, Northwest Ethiopia
Source: PLoS One. 2026 Mar 24;21(3):e0345873. doi: 10.1371/journal.pone.0345873 (PMC13012457; doi:10.1371/journal.pone.0345873)
Supplement: Attachment — Submitted filename: Response_to_Reviewers_auresp_3.docx [file pone.0345873.s005.docx]

**Responses to editors' and reviewers' comment**

First of all, we would like to acknowledge the reviewers and editorial team for giving your time to review this manuscript. Then we have tried to reflect our responses as follow for reviewers and editors according to your concerns.

I. **Editors**

**Additional Editor Comments:**

The abstract should be short and precise. Please revise the background section of your abstract as follows: ‘Surgery and anaesthesia can disrupt normal physiological function through surgical stress and residual anaesthetic effects, increasing the risk of post-anaesthetic complications, known as critical incidents. This study aimed to determine the incidence of critical events in the post-anaesthesia care unit at Debre Markos Comprehensive Specialized Hospital, Ethiopia.” **Response**: corrected as your suggestions
On the results of the abstract section, please provide OR (odds ratio) of associated factors. **Resposne**: corrected
In the conclusion section, you have mentioned that ASA status was associated with the risk of developing critical events. Please be specific about which group of ASA status is associated with complications. **Response**: corrected based on you suggestion
In the conclusion, it is better to mention under/overweight instead of describing them separately. **Response**: corrected
In the introduction section, you don’t need to provide a dearth of information; please be specific to your study and avoid repetition, and try to summarise long statements. **Response**: we have tried to avoid unnecessary repetitions. Thank you.
In the methods section, instead of using the study variable sub-heading, change it to the study outcomes and write it as follows:
The main outcome measure was critical events in the PACU, which was defined as… the point mentioned in the operational definitions, and delete the statement included in the operational definitions of critical outcomes. Then, include the secondary outcomes accordingly. **Response**: we have corrected it
Rewrite your limitations and strengths section as follows: This study's strength lies in its prospective design, which allowed for the comprehensive recording of all complications that occurred during recovery. In contrast, a retrospective study design may overlook critical events or make them difficult to recall. However, a limitation of this study is that it assessed various types of surgical procedures and significant critical event types while encompassing a wide range of age groups. Therefore, it is recommended that further research focus on specific types of surgery. This study was limited to evaluating immediate postoperative complications occurring in the PACU and did not consider delayed yet clinically significant events, such as pulmonary thromboembolism (PTE). Additionally, as this study was conducted at a single institution, conducting research in a multicentre setting with a larger sample size would be beneficial. **Response**: Thank you very much! For your great concerns to strengthen this manuscript for publication. We have done it.

II. **Reviewers**

We are grateful to you for your insightful comments and constructive feedback, which significantly enhance the clarity and rigor of the manuscript.

1. Result: Clarify the first sentence for precision. Instead of "Out of 422 patients, 37.9% of patients developed about 214 complications," consider:

   "Of the 422 patients, 160 (37.9%) experienced one or more critical events, with a total of 214 complications recorded."
    **Response**: we made a correction thank you
   Conclusion: Ensure consistency. The phrase "unhealthy body weight" in the conclusion should be explicitly tied to the BMI categories used (overweight/underweight). **Response**: corrected

   2. Introduction:

   Flow: Improve connectivity between sentences. Use transition words (e.g., Furthermore, Consequently, However) to guide the reader through the rationale.

   Citations: Verify that the statement about limited data in Africa is directly supported by the newly added references (e.g., Blaise Pascal FN et al. 2021). **Response**: we have rechecked and made it appropriate

   3. Methods:

   Ethics: The approval code R/C/S/D/357/01/18 is now included, which is good.

   Clarity: The explanation for not analyzing Clavien-Dindo Grade III-V separately (sample size limitations) is acceptable but should be stated clearly in the limitations section if not already. **Response**: The number of patients with Clavien-Dindo Grade III-V complications is not controllable. But it might be increased and be adequate when originally the sample size is very large. And we mentioned it in a limitation section
   4. Results:

   Language: Replace informal phrases. For example:

   "Even though it’s not surprising..." → "As anticipated, the incidence..." **Response**: we have corrected

   "The proportions... were nearly equal..." → "The proportions were similar across groups, ranging from 35% to 38.4%." **Response**: we made corrections

   Narrative: When discussing Table 5 in the text, explicitly state the key findings (e.g., "Patients attended by untrained nurses had over three times the odds of experiencing a critical event (AOR=3.15...).") Response: we have corrected based on your suggestion.

   5. Discussion:

   Tone: Maintain an objective, scientific tone. Avoid speculative phrasing like "We couldn’t provide the possible reasons..." Instead, state: "The reason for this discrepancy is not clear from our data but may relate to differences in case mix or definitions." **Response**: Thank you dear reviewer for giving us such interesting lesson. Every statement you wrote were meaningful for us. We have corrected

   Structure: When comparing studies, first state the agreement or disagreement, then provide the comparative data, and finally offer a brief, plausible reason for the difference.

**Response**: we have tried our best to make based on your suggestions

6. Language & Grammar (Final Polish):

Conduct a meticulous line-by-line edit focusing on:

Article Use: Ensure correct use of "a," "an," and "the."

Prepositions: Check "in," "on," "at," "for," etc.

Subject-Verb Agreement: (e.g., "The data were analyzed").

Plurals: (e.g., "complications").

Typos: "Sever hypoxemia" → "Severe hypoxemia"; "statistically significant" (not "statically").

**Response:** We have checked the whole document to address the issues of articles, prepositions, grammar, plurals, and typos. We made corrections as much as we can.

7. Figures & Tables:

Flowchart (Figure 1): Ensure it matches the description in the "Response to Reviewers":

Quantify excluded patients (n=11).

Place "n = 422" label next to "Patients included in the study".

Final branch clearly shows: "Patients with ≥1 critical event (n=160)" and "Patients without critical events (n=262)".
 **Response**: we have adjusted based on your suggestions
Submission Checklist: **Response**: We would like to express our sincere gratitude to you who provided us a detailed and constructive feedback through multiple rounds of review. Your valuable comments, professional insights, and shared research experience greatly enhanced the quality, clarity, and scientific rigor of this manuscript.
We have tried to make this manuscript based on this checklist

Before final submission, verify:

The Response to Reviewers letter is complete, polite, and addresses every point raised by each reviewer and the editor.

The Data Availability Statement in the submission system is accurate.

All supporting files (dataset, checklist, ethical approval) are uploaded.

The manuscript text is the "clean" version with all track changes accepted and no comment bubbles.
